# Direct interaction between human DDX1 and SARS-CoV-2 nucleocapsid protein is regulated by phosphorylation

**DOI:** 10.1016/j.jbc.2026.111408

**Published:** 2026-03-26

**Authors:** Liangjun Wang, Ryan M. Baxley, David A. Largaespada, Hideki Aihara, Anja Katrin Bielinsky

**Affiliations:** 1 Department of Biochemistry, Molecular Biology, and Biophysics, University of Minnesota, Minneapolis, Minnesota, USA; 2 Departments of Pediatrics and Genetics, Cell Biology, and Development, University of Minnesota, Minneapolis, Minnesota, USA; 3 Department of Biochemistry and Molecular Genetics, University of Virginia, Charlottesville, Virginia, USA

**Keywords:** DDX1, DDX21, host-pathogen interaction, nucleocapsid protein, protein phosphorylation, protein-protein interaction, RNA helicase, RNA virus, SARS-CoV-2

## Abstract

The nucleocapsid (N) protein of SARS-CoV-2 is essential for viral replication and transcription, in part through interactions with host proteins. Here, we delineate distinct mechanisms underlying N protein association with human RNA helicases DDX1 and DDX21. Co-immunoprecipitation assays in HEK293 cells modified to express N protein revealed that DDX1 binding requires the N protein serine–arginine (SR) region, as SR deletion markedly reduced interaction. Inhibition of glycogen synthase kinase-3 (GSK-3), which targets the SR region, serine-to-alanine substitutions within the SR region, and alkaline phosphatase treatment of extract, respectively, demonstrated that phosphorylation of the SR region is critical for DDX1 binding. Furthermore, phosphorylated or phospho-mimetic SR peptides both prevented N protein-DDX1 complex formation and disrupted preformed complexes *in vitro*, whereas unphosphorylated peptides had no effect, confirming a phosphorylation-dependent binding mechanism. In contrast, interaction with DDX21 was unaffected by SR deletion or phosphorylation status and required both the N- and C-terminal domains of the N protein. RNase treatment enhanced N–DDX21 association without altering N–DDX1 interactions, indicating distinct regulation by RNA. Domain mapping of the two helicases identified the DDX1 N-terminal and the DDX21 C-terminal domains as interfaces that bind the N protein. Together, these findings support phosphorylation-dependent recruitment of DDX1 *versus* phosphorylation-independent engagement of DDX21, highlighting mechanistically distinct strategies by which SARS-CoV-2 N co-opts host helicases.

Severe acute respiratory syndrome coronavirus 2 (SARS-CoV-2) emerged as a novel member of the coronavirus family of RNA viruses in 2019 (1). At the core of the SARS-CoV-2 replication strategy lies the nucleocapsid (N) protein, a multifunctional viral component that is among the most abundant proteins produced during infection and serves as a critical determinant of viral fitness (2, 3). The N protein is essential for encapsulating the single-stranded positive-sense RNA genome, forming helical ribonucleoprotein (RNP) complexes that protect the viral genome, facilitate its transport, and coordinate its replication, transcription, and packaging into progeny virions (2, 4). Additionally, the N protein interacts with other viral proteins, including the membrane (M) protein for virion assembly, and modulates host cellular pathways to evade immune responses and promote pathogenesis (2, 5).

The SARS-CoV-2 N protein is a 419 amino acid polypeptide structurally organized into distinct domains (6, 7). It features an N-terminal domain (NTD) primarily responsible for initial RNA binding through electrostatic interactions, a C-terminal domain (CTD) that mediates oligomerization and additional RNA contacts to stabilize higher-order assemblies, and three intrinsically disordered regions (IDRs): the N-terminal IDR, the central linker region (LKR), and the C-terminal IDR (7). The LKR spans residues 174 to 246 and is noteworthy for its serine-arginine (SR)-rich motif and other charged residues that create a dynamic and modifiable platform (4, 8). The SR region is evolutionarily conserved across coronaviruses and acts as a hotspot for post-translational modifications, most prominently phosphorylation, which fine-tunes the N protein conformation and functional outputs (4, 9).

Phosphorylation of the SR region represents a cornerstone of N protein regulation, orchestrated by a small group of host kinases that hijack cellular signaling pathways to support viral needs (9, 10). Kinases such as serine/arginine-rich protein kinase 1 (SRPK1), glycogen synthase kinase-3 (GSK-3), casein kinase 1 (CK1), cyclin-dependent kinase 1 (CDK-1), and others phosphorylate multiple serine residues within the SR motif in a progressive and hierarchical manner (10, 11). Phosphorylation of the SR region occurs swiftly upon viral entry and peaks during early infection phases (2). These modifications introduce negative charges that alter the charge distribution and hydrophobicity of the N protein, which modulates intra- and intermolecular interactions (10, 12). These changes shift the protein from a compact, RNA-condensing state to an extended, dynamic conformation that adapts to the viral lifecycle demands (7, 10, 12).

In the hypo- or unphosphorylated state, the SARS-CoV-2 N protein forms viscous, gel-like condensates and structured RNP particles through multivalent interactions between its RNA-binding domains and the viral genome (7, 13). This condensed phase promotes genome compaction, shielding it from host nucleases and facilitating incorporation into nascent virions during assembly at the endoplasmic reticulum–Golgi intermediate compartment (10, 13, 14, 15). Conversely, hyperphosphorylation induces a transition to liquid-like droplets *via* liquid-liquid phase separation (LLPS), a biophysical process driven by weakened electrostatic attractions and enhanced solubility (10, 16). These fluid condensates, often colocalizing with viral replication organelles, increase RNA accessibility for the RNA-dependent RNA polymerase (RdRp) complex, thereby boosting transcription of subgenomic RNAs and replication of the full genome (15, 16). Recent studies have shown that this phospho-regulated phase behavior is dynamic, allowing the N protein to cycle between assembly-competent and replication-favoring states as needed (10, 15, 17, 18).

SR region phosphorylation regulates the molecular interactions and subcellular localization of the N protein (19). It disrupts high-affinity RNA-binding interfaces in the NTD and CTD, as well as long-range contacts between domains, reducing non-specific aggregation and aiding in the unpackaging of incoming viral genomes (18). Phosphorylation also creates docking sites for host factors, such as 14-3-3 proteins, which facilitate nucleocytoplasmic shuttling of the N protein, which enables it to interfere with nuclear processes like interferon signaling (20, 21). Evolutionary analyses revealed that mutations enhancing SR phosphorylation confer selective advantages to SARS-CoV-2 variants such as Omicron and Delta by increasing N protein abundance and replication efficiency without impairing RNA packaging or modulation of host protein functions (22, 23).

Coronavirus replication requires the action of RNA helicases. Although coronaviruses encode a viral RNA helicase, nonstructural protein 13 (NSP13), host helicases also contribute to viral replication and gene expression (24). Recent studies suggest that the SARS-CoV-2 N protein interacts with human host DEAD-box (DDX) RNA helicases (25, 26). DDX helicases are an evolutionarily conserved family of proteins that regulate multiple aspects of RNA metabolism, including transcription, translation, splicing, transport, and degradation (27, 28). By catalyzing RNA unwinding and remodeling, they are indispensable for normal cellular function and for the replication of many viruses (27). To date, our understanding of the mechanisms underlying SARS-CoV-2 N protein interactions with host DDX helicases remains incomplete. Two helicases, DDX1 and DDX21 interact with the N protein of coronaviruses, including SARS-CoV-2 (26, 29, 30, 31). DDX1 is generally considered to be proviral, as it facilitates viral replication and transcription (25, 32), whereas DDX21 restricts infection (26). However, a recent study suggested that DDX1 has antiviral activity by interfering with N protein oligomerization through interaction with the N-terminal domain in the N protein (31). In this study, we demonstrate that the SARS-CoV-2 N protein directly interacts with the N-terminal domain of DDX1 through its phosphorylated SR region and that this interaction can be inhibited and disrupted by phosphorylated or phospho-mimetic SR peptides. We also show that N protein engages the CTD of DDX21 through full-length binding in a manner independent of SR phosphorylation. Overall, these data provide valuable insights underlying N protein interactions with human DDX1 and DDX21 helicase, which may be applied to clinical treatment of SARS-CoV-2 infection.

## Results

### The SARS-CoV-2 N protein directly interacts with human DDX1 and DDX21

**Figure 1:**
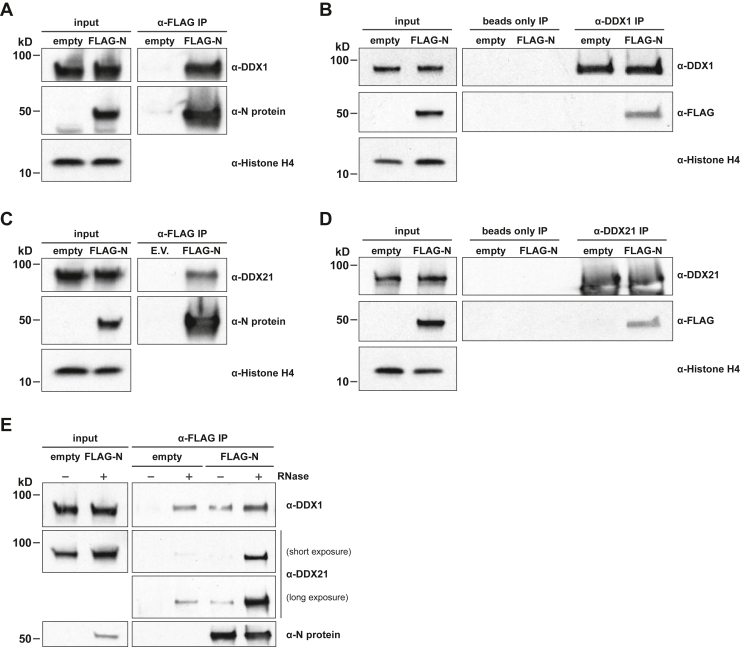
**The SARS-CoV-2 N protein interacts with human DDX1 and DDX21.***A*, Western blot analyses of DDX1 co-immunoprecipitation with FLAG-N protein. Input (*left*) and anti-FLAG immunoprecipitation (IP, *right*) fractions were probed with anti-DDX1, anti-N protein, or anti-Histone H4 antibodies. Histone H4 served as the loading control for input samples. *B*, Western blot analyses of FLAG-N protein co-immunoprecipitation with DDX1. Input (*left*), bead only (*middle*) and anti-DDX1 immunoprecipitation (IP, *right*) fractions were probed with anti-DDX1, anti-FLAG, or anti-Histone H4 antibodies. Histone H4 served as the loading control for input samples. *C*, Western blot analyses of DDX21 co-immunoprecipitation with FLAG-N protein. Input (*left*) and anti-FLAG immunoprecipitation (IP, *right*) fractions were probed with anti-DDX21, anti-N protein, or anti-Histone H4 antibodies. Histone H4 served as the loading control for input samples. *D*, Western blot analyses of FLAG-N protein co-immunoprecipitation with DDX21. Input (*left*), bead only (*middle*) and anti-DDX21 immunoprecipitation (IP, *right*) fractions were probed with anti-DDX21, anti-FLAG, or anti-Histone H4 antibodies. Histone H4 served as the loading control for input samples. *E*, Western blot analyses of DDX1 and DDX21 co-immunoprecipitation in cells expressing empty vector (empty) or full-length FLAG-N protein, with or without RNase digestion as indicated. DDX1, DDX21 and N protein levels in input and anti-FLAG IP samples are shown.

To understand the interaction of the SARS-CoV-2 N protein with human host cell proteins, we overexpressed FLAG-tagged N protein in 293T cells. Utilizing co-immunoprecipitation experiments, we confirmed that the FLAG-N protein pulldown successfully immunoprecipitated human DDX1 and DDX21 helicases (Fig 1, *A* and *C*). Reciprocal immunoprecipitation experiments confirmed that DDX1 and DD21 each co-immunoprecipitated with FLAG-N protein, respectively (Fig. 1, *B* and *D*). Because DDX1, DDX21, and SARS-CoV-2 N protein are each RNA-binding proteins, we wanted to determine the contribution RNAs may make to protein-protein interaction. To this end, we treated total cell extracts with RNase to degrade RNAs and then investigated the effects on N protein-DDX1 and N protein-DDX21 immunoprecipitation. The results showed that RNA digestion only modestly affects N protein interaction with DDX1 under these conditions (Fig. 1*E*). However, N protein binding to DDX21 was markedly increased after RNAse treatment. Our data indicate that eliminating RNA causes a low level of non-specific enrichment for both DDX1 and DDX21. These findings suggest that RNA suppresses non-specific interactions with the anti-Flag affinity beads, but it does not mediate the interaction between the N protein and DDX1 or DDX21, respectively.

**Figure 2:**
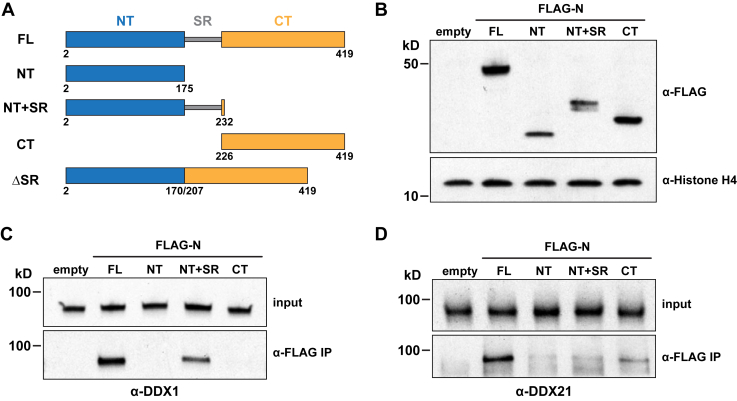
**Mapping the N protein domains responsible for binding to DDX1 and DDX21.***A*, Cartoon schematic of full-length and truncated SARS-CoV-2 N protein constructs. *B*, Western blots analysis of N protein expression levels in cells expressing empty vector (empty), full-length (FL) and truncated FLAG-N proteins (NT, NT+SR, CT) detected by anti-FLAG antibodies with anti-Histone H4 used as the loading control. *C*, Western blot analyses of DDX1 co-immunoprecipitation in cells expressing empty vector (empty), full-length (FL) and truncated FLAG-N proteins (NT, NT+SR, CT). DDX1 levels in the input and anti-FLAG IP samples are shown. *D*, Western blot analyses of DDX21 co-immunoprecipitation in cells expressing empty vector (empty), full-length (FL) and truncated FLAG-N proteins (NT, NT+SR, CT). DDX21 levels in the input and anti-FLAG IP samples are shown.

To map the N protein domains responsible for interaction with DDX1 or DDX21, we designed constructs for defined N protein deletion mutants (Fig. 2*A*; NT, NT+SR, CT) and confirmed their expression in 293T cells (Fig. 2*B*). Next, we performed FLAG-N protein immunoprecipitation experiments and found that DDX1 interacted with FL and NT+SR, but not NT and CT FLAG-tagged N protein fragments. These data suggest that the SR region is critical to the DDX1/N protein interaction. In parallel, we used the same N protein fragments to investigate interaction with DDX21. We found that robust DDX21 immunoprecipitation only occurs in the presence of full-length N protein (Fig. 2*D*).

### Mapping the SARS-CoV-2 N protein interaction domains of human DDX1 and DDX21

**Figure 3:**
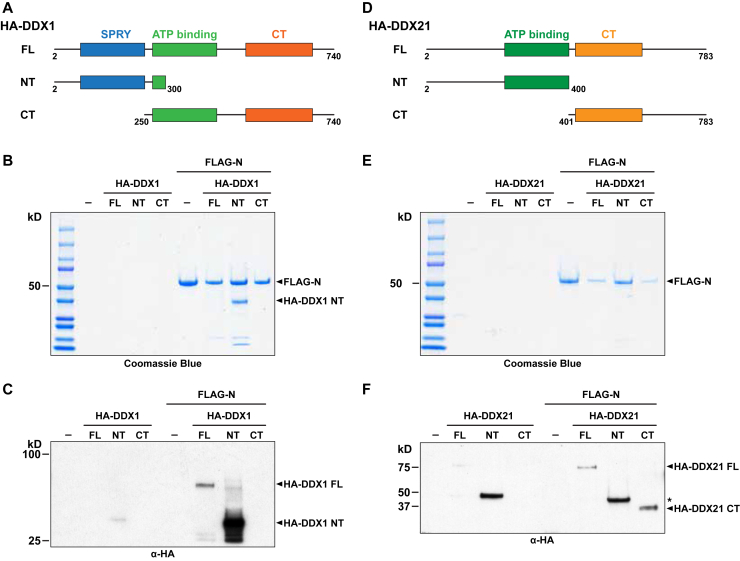
**Mapping the DDX1 and DDX21 domains responsible for binding to N protein.***A*, schematic representation of DDX1 protein constructs, including full-length (FL), N-terminal (NT) or C-terminal (CT) fragments. The SPRY (*blue*), ATP binding (*green*) and CT (*orange*) domains are depicted. *B* and *C*, Coomassie blue staining (*B*) and anti-HA tag Western blot analyses (*C*) for proteins immunoprecipitated with anti-FLAG beads. Samples include no HA-tagged DDX1 (−) or full-length, NT or CT fragments, as well as FLAG-N protein. *D*, schematic representation of DDX21 protein constructs, including full-length (FL), N-terminal (NT) or C-terminal (CT) fragments. The ATP-binding (*green*) and CT (*orange*) domains are depicted. *E* and *F*, Coomassie blue staining (*E*) and anti-HA tag Western blot analyses (*F*) for proteins immunoprecipitated with anti-FLAG beads. Samples include no HA-tagged DDX21 (−) or full-length, NT or CT fragments, as well as FLAG-N protein.

To understand the DDX1 and DDX21 protein domains that contributed to interaction with the SARS-CoV-2 N protein, we used a baculovirus expression system to express FLAG-N as well as HA-tagged DDX1 or DDX21. For DDX1, the full-length protein containing the SPRY, ATP-binding, and CTD and fragments including just the N-terminal or C-terminal portion of the protein (Fig. 3*A*) were expressed alone or co-expressed with FLAG-N protein. Protein complexes were purified using anti-FLAG beads and analyzed with Coomassie staining (Fig. 3*B*), and the results were confirmed using Western blot analyses (Fig. 3*C*). These data showed that the N-terminal fragment of HA-DDX1 robustly copurified with FLAG-N protein, whereas full-length HA-DDX1 copurified less efficiently and was only detectable by Western blot. This was not due to differences in expression levels, as the input controls show that full-length protein is more highly abundant than the N-terminal fragment of the protein (Fig. S1). For DDX21, the full-length protein containing the ATP binding and CTD and fragments including just the N- or C-terminal portion of the protein (Fig. 3*D*) were expressed alone or co-expressed with the FLAG-N protein. As with DDX1, Coomassie staining (Fig. 3*E*) and Western blot analyses (Fig. 3*F*) were used to analyze copurified proteins. These results showed that none of the HA-DDX21 fragments was visible by Coomassie staining, but all three fragments were detectable by Western blot. Importantly, the N-terminal fragment of HA-DDX21 was also significantly enriched in the nonspecific pulldown control lane. Overall, these data confirm both the DDX1-and DDX21-N protein interactions and provide insight into the regions of each protein important for binding to the SARS-CoV-2 N protein.

### Phosphorylation of the SR region is required for N protein interaction with human DDX1

**Figure 4:**
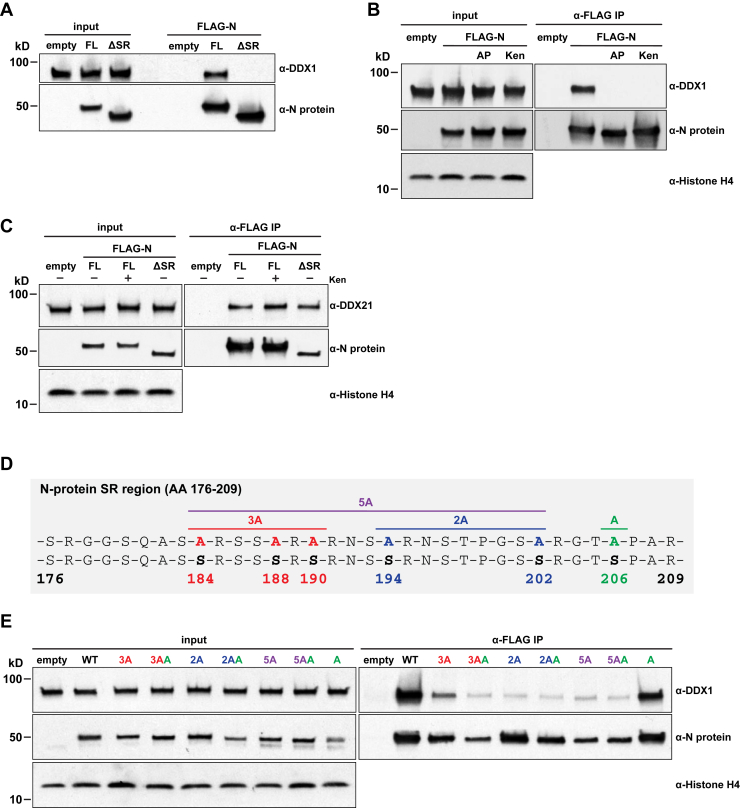
**Dephosphorylation and mutation of SR region reduces N protein interaction with DDX1.***A*, Western blot analyses of DDX1 co-immunoprecipitation in cells expressing empty vector (empty), full-length (FL) or ΔSR FLAG-N proteins. DDX1 and N protein levels in the input and anti-FLAG IP samples are shown. *B*, Western blot analyses of DDX1 co-immunoprecipitation in cells expressing empty vector (empty) or FLAG-N protein. For “AP” samples, extracts were treated with alkaline phosphatase. For “Ken” samples, cells were treated with kenpaullone, a GSK-B inhibitor. DDX1, N protein and Histone H4 levels in input and anti-FLAG IP samples are shown. *C*, Western blot analyses of DDX21 co-immunoprecipitation in cells expressing empty vector (empty), full-length (FL) or ΔSR FLAG-N proteins. For “Ken” samples, cells were treated with kenpaullone, a GSK-3 inhibitor. DDX21, N protein and Histone H4 levels in input and anti-FLAG IP samples are shown. *D*, schematic representation of the N protein SR region, including the serine (S) to alanine (A) mutation constructs tested. *E*, Western blot analyses of DDX1 co-immunoprecipitation in cells expressing empty vector (empty), wild type (WT) or alanine mutant FLAG-N proteins. Details of the alanine mutants can be found in *panel A*. DDX1, N protein and Histone H4 levels in input and anti-FLAG IP samples are shown. A replicate of this experiment is shown in Fig. S2.

Our structure-function analysis suggested that the SR region of the SARS-CoV-2 N protein (Fig. 2*C*) is critical for its interaction with DDX1. To confirm this observation, we repeated immunoprecipitation experiments using extracts from cells expressing either empty vector, full-length or ΔSR N protein constructs (Fig. 4*A*). These data demonstrated that the N protein SR region is essential for interaction with human DDX1. The phosphorylation of this region has been implicated in the regulation of N protein functions during viral replication, transcription, assembly and packaging (10, 15, 32). To assess whether N protein phosphorylation affects the DDX1 interaction, we treated cell extracts with alkaline phosphatase (AP) to abolish all phosphorylation. In parallel, cells were treated with the GSK-3B inhibitor Kenpaullone, as GSK-3B was previously linked to SR region phosphorylation (32). Interestingly, both treatments completely disrupted co-immunoprecipitation of DDX1 with FLAG-N protein (Fig. 4*B*). Unlike the DDX1 interaction, neither Kenpaullone treatment nor complete deletion of the SR region affected DDX21 binding to N protein (Fig. 4*C*). To extend our analyses of SR region phosphorylation, we engineered seven different N protein constructs containing single or varying combinations of serine (S) to alanine (A) substitutions (Fig. 4*D*) for expression in 293T cells. Immunoprecipitation experiments showed that the inability to robustly phosphorylate the N protein SR region significantly reduced the interaction with DDX1 (Fig. 4*E*, replicate experiment shown in Fig. S2). Taken together, these data clearly demonstrate the importance of the phosphorylated N protein SR region to mediate protein-protein interaction with human DDX1.

### Phosphorylated and phospho-mimic SR region peptides disrupt DDX1-N protein binding

**Figure 5:**
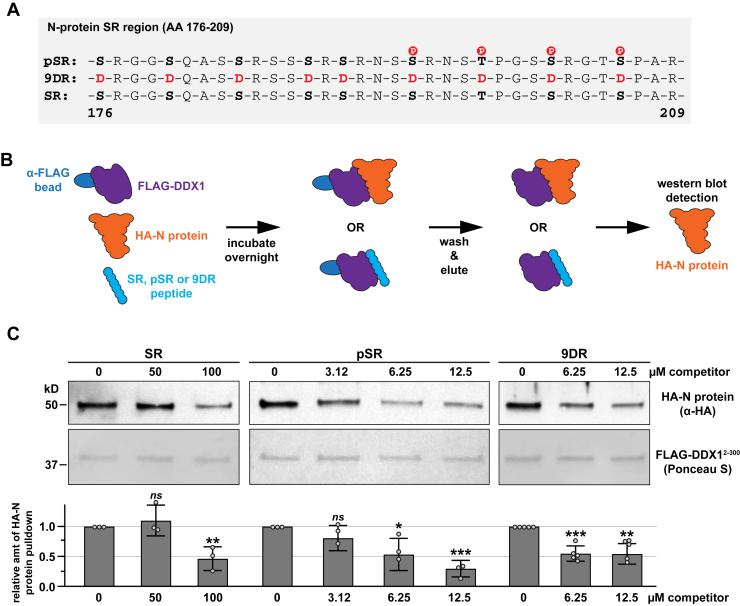
**SR region peptides compete with full-length N-protein for binding to DDX1^2-300^.***A*, schematic representation of the N protein SR region including the phospho-SR (pSR) and serine (S) to aspartate (D) mutation (9DR) constructs tested. *B*, schematic cartoon of the *in vitro* protein interaction competition assay. *C*, Ponceau S staining and anti-HA tag Western blot analyses of the *in vitro* binding between HA-N protein and FLAG-DDX1^2-300^ in the presence or absence of peptide competitors including the N-protein SR region (SR), phospho-SR (pSR) and serine to aspartate mutation (9DR) peptides. The relative amount of HA-N pulldown normalized to the Ponceau S loading control for each condition is graphed with the no peptide controls set to 1. Individual data points representing biological replicates are displayed as *gray circles* and error bars indicate standard deviation. Statistical significance was calculated using an unpaired, two-tailed student’s *t* test with ∗∗∗ < 0.001, ∗∗ < 0.005, ∗ < 0.05 and *ns* = not significant.

**Figure 6:**
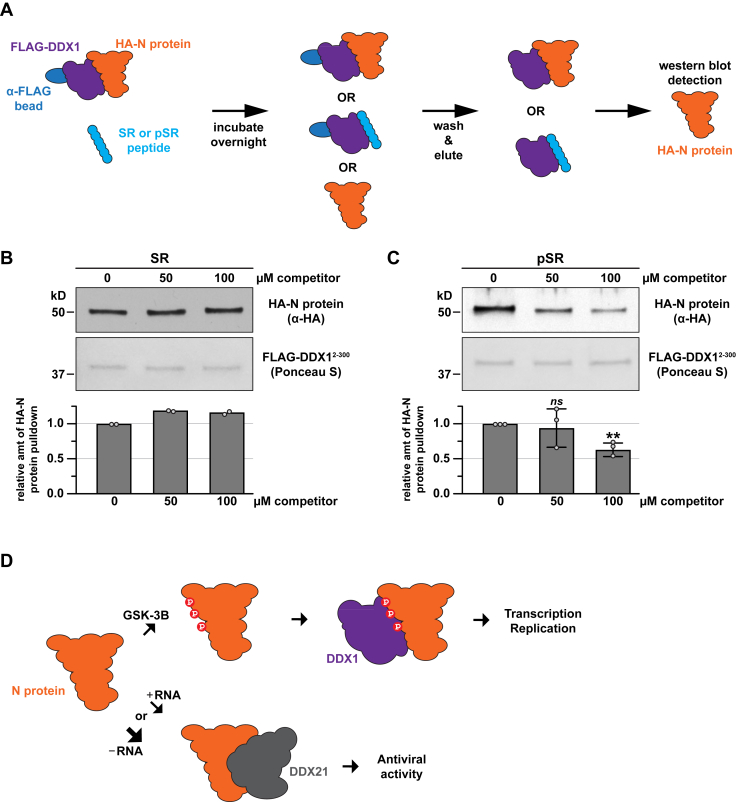
**The phospho-SR (pSR) region peptide disrupts the interaction between full-length N-protein and DDX1^2-300^.***A*, schematic cartoon of the *in vitro* protein interaction disruption assay. *B*, Ponceau S staining and anti-HA tag Western blot analyses of the *in vitro* binding between HA-N protein and FLAG-DDX1^2-300^ in the presence or absence of the SR peptide competitor. The relative amount of HA-N pulldown normalized to the Ponceau S loading control for each condition is graphed with the no peptide controls set to 1. Individual data points representing biological replicates are displayed as *gray circles*. *C*, Ponceau S staining and anti-HA tag Western blot analyses of the *in vitro* binding between HA-N protein and FLAG-DDX1^2-300^ in the presence or absence of the phospho-SR (pSR) peptide competitor. The relative amount of HA-N pulldown normalized to the Ponceau S loading control for each condition is graphed with the no peptide controls set to 1. Individual data points representing biological replicates are displayed as *gray circles* and error bars indicate standard deviation. Statistical significance was calculated using an unpaired, two-tailed student’s *t* test with ∗∗ < 0.005 and *ns* = not significant. *D*, cartoon summary the data describing N-protein interactions. (*Top*) The SR region of the SARS-CoV-2 N protein is phosphorylated by GSK-3B which promotes interaction with DDX1 to promote viral transcription and replication. (*Bottom*) The SARS-CoV-2 N protein can also interact with DDX21, which is more robust in the absence of RNA binding, to promote antiviral activity.

To investigate the DDX1-N protein interaction further, we determined whether synthesized N protein SR region peptides could disrupt this interaction *in vitro*. Using current technology, a maximum of four serine or threonine residues can be *in vitro* phosphorylated; therefore, alongside a phosphorylated (pSR) peptide, we also synthesized phosphomimetic peptides carrying alanine to aspartate substitutions (4DR, 9DR; Figs. 5*A* and S3*A*). First, we used a competition assay depicted in Figure 5*B* to examine how each peptide affected the DDX1-N protein interaction. These data showed the 50% inhibition concentrations for both the pSR and 9DR peptides were ∼16-fold lower than for the SR peptide (∼6.25 μM *versus* ∼100 μM, respectively; Fig. 5*C*). These data suggest that unmodified SR peptides can interact with N protein in our *in vitro* system, albeit with significantly lower affinity than phosphorylated or phosphomimetic SR peptides. However, the phosphomimetic 4DR peptide did not significantly reduce DDX1-N protein interaction at the concentrations tested (Fig. S3*B*). Because the 4DR and pSR peptides carry phosphorylated S/T or phoshomimetic D substitutions at the same residues, our findings suggest that phosphorylated peptides provide more robust binding activity in comparison to phosphomimetic peptides. Next, we performed similar assays using preformed DDX1-N protein complexes and assessed whether the SR peptides could disrupt their association (Fig. 6*A*). While non-phosphorylated SR peptide did not disrupt preformed complexes (Fig. 6*B*), ∼40% of DDX1-N protein complexes were disrupted with the addition of 100 μM pSR (Fig. 6*C*). Taken together, these data provide important insights into specific post-translational modifications of the SARS-CoV-2 N protein SR region important for binding to human DDX1.

## Discussion

The SARS-CoV-2 N protein orchestrates multiple steps of the viral life cycle, coordinating genome packaging and replication while engaging host factors, including the human RNA helicases DDX1 and DDX21, to modulate cellular pathways (2, 26). In contrast to a previous report (31), our findings reveal that the N protein SR region associates with the N-terminal domain of DDX1 in a phosphorylation-dependent manner. This interaction is disrupted by deletion of the SR region, phosphorylated or phosphomimetic SR peptides, while alanine substitutions of serine residues substantially reduce binding. In contrast, the SR region is dispensable for association with the CTD of DDX21, as SR motif deletions exert no detectable effect. RNA depletion enhanced N–DDX21 binding but had no influence on N–DDX1 association, underscoring distinct modes of regulation (Fig. 6*D*).

The phosphorylation reliance of N–DDX1 binding parallels established mechanisms in other coronaviruses, where N protein phosphorylation facilitates helicase recruitment to support viral RNA synthesis (32). Our results extend this paradigm to SARS-CoV-2, highlighting the SR region as a phosphorylatable hub targeted by host kinases such as GSK-3 (11, 32). Inhibition of GSK-3 or alkaline phosphatase pretreatment abolished N–DDX1 binding, consistent with the interaction being phosphorylation-dependent (32). Our mutagenesis data reinforce the phosphorylation dependence of N–DDX1 association. Serine-to-alanine substitutions across the SR region, encompassing five putative GSK-3 sites and the CDK1 site at S206, substantially reduced binding, consistent with prior reports linking SR mutations to impaired N oligomerization, RNA binding, and replication (10, 15, 18, 33, 34). Different serine mutation combinations produced comparable reductions, indicating that multiple residues collectively contribute and that the SR region functions as an integrated phosphorylation hub rather than being dominated by a single site. Notably, mutation of the putative priming site S206 reduced N–DDX1 binding by 50% to 70%, suggesting that this residue facilitates efficient phosphorylation cascades but is not strictly essential, as additional serines can partially compensate in its absence (10, 11).

Our results reveal a differential role of authentic phosphorylation versus phosphomimetic substitutions in modulating N–DDX1 complex formation. Both a four-serine phosphorylated SR peptide (pSR) and a nine-serine-to-aspartate substitution peptide (9DR) were able to block complex assembly with purified N protein at low micromolar concentrations, indicating that introducing a strong negative charge within the SR region effectively competes with unmodified N–DDX1 engagement. By contrast, the peptide (4DR), in which aspartates replaced only four phosphorylated serines, failed to inhibit complex formation. This distinction underscores that aspartate substitutions approximate charge but do not fully reproduce the steric bulk, hydrogen-bonding capacity, or conformational impact of phosphorylated serines (35). Importantly, the phosphorylated peptides were uniquely able to disrupt preformed N–DDX1 complexes *in vitro*. This finding suggests that authentic phosphorylation not only prevents complex formation but also actively destabilizes established interactions, likely through strong electrostatic repulsion or direct competition at binding surfaces. The disruptive effects of phosphorylated peptides suggest competitive or allosteric inhibition, whereby phosphorylation alters the SR region’s electrostatics or conformational flexibility, thereby preventing DDX1 docking (36, 37, 38, 39).

Together, these observations support a model in which phosphorylation of the SR region operates as a reversible molecular switch, controlling both the recruitment and release of DDX1 during distinct stages of the viral life cycle. This emphasizes the need to study authentic phosphorylation events, as phosphomimetic substitutions cannot fully replicate the dynamic regulatory potential of phosphorylated serines. This phosphorylation switch may therefore regulate the temporal recruitment of DDX1, enabling helicase-mediated unwinding of double-stranded RNA intermediates that accumulate during replication (10, 17, 32).

Our results diverge from those reported by Min *et. al*., possibly reflecting differences in assay conditions (*e.g*., overexpression levels) and cell line properties. The authors mapped DDX1 binding mainly to the N-terminus of the N protein, reporting that the interaction persists even without the SR region and functions as an antiviral restriction mechanism by blocking N protein oligomerization and viral replication (31). Several factors may contribute to this discrepancy. First, differences in affinity tags can affect which interactions are recovered: FLAG tags (our study) generally yield higher purity, whereas streptavidin tags (Min *et al.*) provide high specificity but may introduce elution-related artifacts (40, 41). In addition, the pCAGGSP7 plasmid used by Min *et al.* typically drives higher and more uniform expression across diverse cell types than the pcDNA3.1 vector used in our experiments (42, 43, 44). Under excessively high expression conditions, the N protein may not become fully phosphorylated, which can increase the proportion of non-physiological, non-specific interactions. Third, cellular context matters, as kinase activity and other regulatory pathways differ across cell types and can shift the balance between phosphorylation-dependent and phosphorylation-independent interactomes (15, 45). Resolving these differences will require matched experimental conditions and systematic comparison of both phospho-dependent and phospho-independent DDX1–N interactions across multiple assays and cellular models (26, 30, 31, 32).

Our findings have important therapeutic implications as the SARS-CoV-2 N protein SR region emerges as a vulnerability within the viral replication machinery. Inhibition of SRPK1 or GSK-3 diminishes N protein phosphorylation, disrupts liquid–liquid phase separation, and impairs ribonucleoprotein assembly (11, 32). Together with our data, this suggests that targeting kinase-mediated phosphorylation or N protein-helicase interfaces may destabilize N–helicase complexes and suppress coronavirus replication, including emerging variants. While the S protein is the most frequently mutated SARS-CoV-2 viral protein, variant analyses readily detect changes within the N protein (46). Notably, the SR region (aa 164–205) is a hotspot for N protein mutations (46). Serine residues that are targeted for phosphorylation are infrequently mutated; rather, R203 and G204 represent the most mutated amino acid positions (23, 46, 47, 48). Although these residues are not directly phosphorylated, amino acid substitutions at these residues alter the pattern of SR region phosphorylation in cell culture models (23, 34, 48). Therefore, future monitoring and analyses of SR region variants may provide valuable insight into the potential value of targeting N protein function therapeutically (48).

In contrast to the N protein interaction with DDX1, N binding to the CTD of DDX21 is independent of the SR region, indicating a distinct mechanism of engagement. DDX21 is a nucleolar helicase with established functions in ribosome biogenesis, RNA processing, and innate immune signaling (27, 49, 50). Our results indicate that the N protein associates with DDX21 primarily through its structured domains, consistent with studies showing N protein dimers adopting extended conformations to mediate protein–protein contacts in RNA-free states (51). Unlike the dynamically regulated N–DDX1 interaction, the phosphorylation-independent N–DDX21 association may be constitutive or intrinsically more stable than N-DDX1 binding. Given DDX21’s reported role in double-stranded RNA sensing and interferon induction, N protein binding may suppress host antiviral signaling by sequestering DDX21 away from immune sensors (49, 50). Small molecules that disrupt this interaction could therefore restore host antiviral responses.

The enhancement of N-DDX21 binding upon RNA removal implies that RNA acts as a competitive modulator, possibly sterically hindering the interface or stabilizing alternative N conformations that favor RNA over protein binding (51, 52, 53). Contrary to N-DDX1, where interactions persist in RNA-bound states—potentially during active transcription—the N-DDX21 complex, which is more stable in the absence of RNA may be optimized for post-replication phases, such as virion assembly or evasion of host RNA decay pathways. This differential regulation could enable SARS-CoV-2 to exploit DDX21 for suppressing innate immunity, as evidenced by N protein-mediated disruption of stress granules, while reserving DDX1 for RNA remodeling (25, 26, 32, 54).

Broader interactome analyses reinforce these distinctions, positioning DDX1 and DDX21 within a network of host factors co-opted by N protein (25, 26, 30). We propose a model (Fig. 6*D*) in which phosphorylation and RNA-binding dynamically partition N protein into distinct functional states: RNA-bound and phosphorylated N protein preferentially recruits DDX1 to promote replication, whereas RNA-free N protein may shift toward DDX21 engagement, facilitating antiviral activities potentially by N protein sequestration to the nucleolus (55), which would provide a mechanism for restricting infection and escaping the innate immune response (Fig. 6*D*). These mutually exclusive interactions underscore the versatility of the N protein in coordinating replication and immune evasion (13, 25, 26, 30, 32, 56).

In addition to its interactions with DDX1 and DDX21, the SARS-CoV-2 N protein engages a diverse array of host factors to modulate immune responses, promote viral replication, and drive pathogenesis (57). For example, it binds to RIG-I (retinoic acid-inducible gene I), repressing interferon-β production and impairing IRF3 (interferon regulatory factor 3) phosphorylation, which facilitates immune evasion (57, 58). Similarly, associations with TRIM25 (tripartite motif containing 25) inhibit RIG-I-mediated interferon signaling, while interactions with TBK1 (TANK-binding kinase 1) prevent IRF3 nuclear translocation and further suppress antiviral immunity (57, 58). The N protein also sequesters G3BP1 (Ras-GTPase-activating protein-binding protein 1) to disrupt stress granule formation and inhibit type I interferon production, and it engages MAVS (mitochondrial antiviral signaling protein) to block its polyubiquitination and aggregation, thereby attenuating downstream signaling cascades (57, 59). Furthermore, it promotes NLRP3 (NLR family pyrin domain containing 3) inflammasome assembly by directly binding NLRP3 and enhancing its interaction with ASC (apoptosis-associated speck-like protein containing a caspase recruitment domain), leading to proinflammatory cytokine release, yet it suppresses pyroptosis by preventing Gasdermin D cleavage (57, 60). Additional engagements include activating NF-κB (nuclear factor kappa-light-chain-enhancer of activated B cells) pathways through TLR2 (Toll-like receptor 2), TAK1 (transforming growth factor-beta-activated kinase 1), and IKK (IκB kinase) recruitment, as well as binding RAGE (receptor for advanced glycation end products) to stimulate ERK1/2 (extracellular signal-regulated kinases 1 and 2)-NF-κB signaling, and exacerbate lung inflammation (57, 61). These interactions highlight the N protein’s versatility in hijacking host machinery, suggesting broader therapeutic strategies targeting these interfaces to mitigate COVID-19 severity.

Despite the advances in understanding N protein interactions presented here, key limitations remain. Our reliance on overexpression, co-immunoprecipitation, and *in vitro* assays may not fully capture physiological context, including compartmentalization or cofactors that influence these interactions *in vivo*. Moreover, the precise identity of the kinases involved, their relative contributions, and the timing of SR phosphorylation during the viral life cycle (*e.g.*, replication vs. assembly) remain unresolved (10, 11, 32). Future studies employing infectious clones, CRISPR-edited host cells, and live-cell imaging will be critical for validating these mechanisms and clarifying their contributions to viral pathogenesis.

In summary, we delineate two distinct binding paradigms: phosphorylation-dependent recruitment of DDX1 and phosphorylation-independent engagement of DDX21 by the SARS-CoV-2 N protein (Fig. 6*D*). These differential binding modes emphasize the adaptability of N protein as a central hub integrating replication and immune modulation. These findings identify the SR region and N–helicase interfaces as attractive therapeutic targets, offering avenues for disrupting coronavirus replication at multiple mechanistic levels (11, 45, 47, 62).

## Experimental procedures

### Cell lines

HEK293T (ATCC Cat# CRL-3216, RRID:CVCL_0063) cells were cultured in Dulbecco’s Modified Eagle Medium (DMEM medium Gibco 11995-065) supplemented with 10% fetal bovine serum (Sigma F4135), 1% Penicillin- Streptomycin (Gibco 15140), and 5% CO2 at 37 °C. Sf9 (Thermo Fisher 11496015) cells were cultured in Sf-900 II SFM (Gibco 10902-088) at 28 °C. Cells used in this study were authenticated using morphology checks by microscope and *mycoplasma* testing.

### Generation and site-directed mutagenesis of plasmid constructs

Coding sequences for the full-length and truncated SARS-CoV-2 N protein fragments were amplified from pLVX-EF1alpha-SARS-CoV-2-N-2xStrep-IRES-Puro (RRID:Addgene_141391). Coding sequences for full-length and truncated DDX1 or DDX21 protein fragments were amplified from GenScript plasmid OHu15746 or p23-DDX21 WT (RRID:Addgene_128803). During PCR amplification 5′ FLAG or HA tag sequences were added, and products were cloned into a pcDNA vector (Addgene V790–20). Site-directed mutagenesis of plasmids expressing full-length and truncated SARS-CoV-2 N protein fragments was performed using QuickChange XL site-directed mutagenesis (Agilent Cat# 200517) according to the manufacturer's protocol. Mutations were confirmed by PCR amplification and Sanger sequencing. Primer sequences are available upon request.

### Cell transfection and protein extract preparation

SARS-CoV-2 N protein pcDNA constructs (full-length, truncated, site-directed mutations) were transfected into HEK293T cells using FuGENE HD transfection reagent (Promega Cat# E2311) according to the manufacturer’s protocol. Cells were collected 48 h post-transfection and resuspended in extraction buffer (50 mM Tris-HCl, pH 8.0, 100 mM NaCl, 10 mM NaF,10% Glycerol, and 1 mM DTT, with protease inhibitors). The next samples were frozen/thawed three times and then briefly sonicated five times.

### SARS-CoV-2 N protein pulldown

Total cell extracts (150 μg) were incubated with 25 μl anti-Flag affinity beads (Bimake Cat# B23102, RRID:AB_2728745) in 200 μl IP150 buffer (10 mM Hepes pH 8.0, 150 mM NaCl, 10 mM NaF, 10% glycerol, 0.1% triton-X100) at 4 °C overnight. The beads were washed as follows: once with IP150, once with IP300 (10 mM Hepes pH 8.0, 300 mM NaCl, 10 mM NaF, 10% glycerol, 0.1% triton-X100), three times with IP500 (10 mM Hepes pH 8.0, 500 mM NaCl, 10 mM NaF, 10% glycerol, 0.1% triton-X100), once with IP300, and finally once with IP150. Interacting proteins were eluted with 30 μl of 0.1 M glycine pH 2.2. Next, 6 μl of 0.5 M Na_2_HPO_4_ pH 8.3 was added to neutralize the eluted materials. One-third of each elution was used for Western blot analyses. For dephosphorylation experiments, total cell extracts were treated with 5 units of alkaline phosphatase (NEB Cat# M0371L) at 37 °C for 1 h, or cells were pretreated with 2 μM kenpaullone (Tocris Cat# 1398) for 48 h prior to transfection with pcDNA-Flag-N constructs. For RNase treatment experiments, total cell extracts (150 μg) were treated with 1 μg RNase (Thermo Fisher EN0531) at 37 °C for 1 h.

### Baculovirus protein expression

Full-length or truncated cDNAs encoding the SARS-CoV-2 N protein, human DDX1, or human DDX21 were inserted into pFastBac1 and expressed in insect Sf9 cells using the Bac-to-Bac system (Thermo Fisher Scientific 10360014) according to the manufacturer's protocol. Protein purifications were performed as described previously (63). Primer sequences are available upon request.

### Western blot analyses

Samples were separated on Mini-Protean TGX precast gels (BioRad Cat# 4561086 or 4561084) and transferred to nitrocellulose membranes (BioRad Cat# A29668365) and blocked with 5% non-fat milk in TBST. Primary antibodies used for Western blot detection include anti-N protein (ProSci Cat# 9103; 1:1000), anti-Flag M2 antibody (Sigma Cat# F3165, RRID:AB_259529; 1:2000), anti-DDX1 (Proteintech Cat# 11357-1-AP, RRID:AB_2092222; 1:1000), anti-DDX21 (Proteintech Cat# 10528-1-AP, RRID:AB_2092705; 1:5000), and anti-H4 (Abcam Cat# ab7311, RRID:AB_305837; 1:100000). Secondary antibodies used for Western blot detection include goat anti-rabbit (Jackson ImmunoResearch Labs Cat# 111-035-144, RRID:AB_2307391) and goat anti-mouse (Jackson ImmunoResearch Labs Cat# 115-035-003, RRID:AB_10015289) at 1:10,000. When possible, the specificity of antibodies used was confirmed used negative (empty vector) controls. Detection was performed using WesternBright Quantum detection (K-12042-D20), and densitometry was performed using the FIJI (v.2.16.0/154p; RRID:SCR_002285) and Microsoft Excel (RRID:SCR_016137).

### Protein interaction competition and disruption assays

For competition assays, anti-Flag affinity beads (Bimake Cat# B23102, RRID:AB_2728745) were incubated with FLAG-DDX1^(2-300)^ (10 μM), HA-N protein (10 μM), and N protein SR region peptides (See Fig. 5*A*) (0–100 μM, as indicated in Fig. 5) in 200 μl IP 150 buffer with 2% BSA at 4 °C overnight. Next, beads were washed as described above for SARS-CoV-2 N protein pulldown, protein complexes were eluted with 0.8 mg/ml Flag peptide, and separated on Mini-Protean TGX precast gels (BioRad Cat# 4561086 or 4561084) and transferred to nitrocellulose membranes (BioRad A29668365), and stained with 0.1% Ponceau S (Sigma P3504) to show the amount of FLAG-DDX1^2-300^, then analyzed HA-N protein using Western blot as described above. Disruption assays were performed as described for competition assays with the following modifications. First, anti-Flag affinity beads were incubated with FLAG-DDX1^(2-300)^ (10 μM) and HA-N protein (10 μM) for 2 h at 4 °C to allow formation of beads-DDX1/N-protein complexes. Next, samples were incubated with 0 to 100 μM N protein SR/pSR peptide (as indicated in Fig. 6) overnight. Samples were then washed, eluted, and analyzed as described above.

## Data availability

The data presented in this study are included in the article and supporting information. The raw data supporting the conclusions of this article will be made available by the authors upon request. Please address requests to Anja-Katrin Bielinsky (azu3jn@virginia.edu).

## Supporting information

This article contains supporting information.

## Conflict of interest

A. K. B. is an Editorial Board Member for this journal and was not involved in the editorial review or the decision to publish this article. D. A. L. is the co-founder and co-owner of several biotechnology companies including NeoClone Biotechnologies, Inc, Discovery Genomics, Inc (recently acquired by Immusoft, Inc), B-MoGen Biotechnologies, Inc (recently acquired by Bio-Techne Corporation), and Luminary Therapeutics, Inc. D. A. L. holds equity in, serves as a Senior Scientific Advisor for and Board of Director member for Recombinetics, a genome editing company. D. A. L. consults for Genentech, Inc, which is funding some of his research. The business of all these companies is unrelated to the contents of this manuscript.
