# Supplementary material for: Direct interaction between human DDX1 and SARS-CoV-2 nucleocapsid protein is regulated by phosphorylation
Source: J Biol Chem. 2026 Mar 26;302(5):111408. doi: 10.1016/j.jbc.2026.111408 (PMC13125193; doi:10.1016/j.jbc.2026.111408)
Supplement: Supplementary material [file mmc1.pdf]

Figure S1

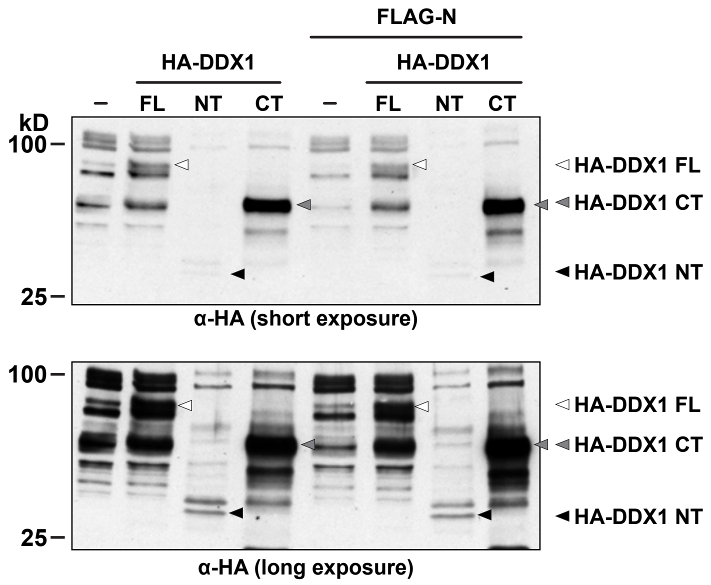

Figure S1. Input controls for copurification of baculovirus expressed FLAG-N protein with HA-DDX1 fragments.

Input controls corresponding to Figure 3C. Anti-HA tag western blot analyses for input samples including no HA-tagged DDX1 (-) or full-length, NT or CT fragments, with or without co-expression of FLAG-N protein.
