# Supplementary material for: Direct interaction between human DDX1 and SARS-CoV-2 nucleocapsid protein is regulated by phosphorylation
Source: J Biol Chem. 2026 Mar 26;302(5):111408. doi: 10.1016/j.jbc.2026.111408 (PMC13125193; doi:10.1016/j.jbc.2026.111408)
Supplement: Supplementary material [file mmc2.pdf]

Figure S2

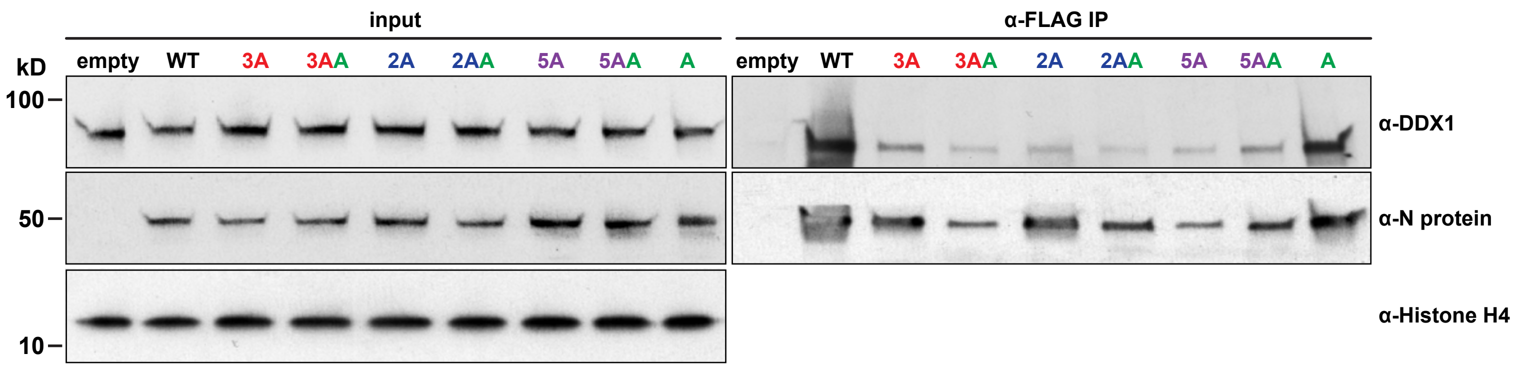

Figure S2. Mutation of SR region reduces N protein interaction with DDX1.
Replicate of the experiment shown in Figure 4E. Western blot analyses of DDX1 co-immunoprecipitation in cells expressing empty vector (empty), wild type (WT) or alanine mutant FLAG-N proteins. Details of the alanine mutants can be found in panel A. DDX1, N protein and Histone H4 levels in input and anti-FLAG IP samples are shown.
