# Supplementary material for: Direct interaction between human DDX1 and SARS-CoV-2 nucleocapsid protein is regulated by phosphorylation
Source: J Biol Chem. 2026 Mar 26;302(5):111408. doi: 10.1016/j.jbc.2026.111408 (PMC13125193; doi:10.1016/j.jbc.2026.111408)
Supplement: Supplementary material [file mmc3.pdf]

Figure S3

A

N-protein SR region (AA 176-209)

```
4DR: -S-R-G-G-S-Q-A-S-S-R-S-S-S-R-S-R-N-S-D-R-N-S-D-P-G-S-D-R-G-T-D-P-A-R-
SR:  -S-R-G-G-S-Q-A-S-S-R-S-S-S-R-S-R-N-S-S-R-N-S-T-P-G-S-S-R-G-T-S-P-A-R-
     176                                                                209
```

B

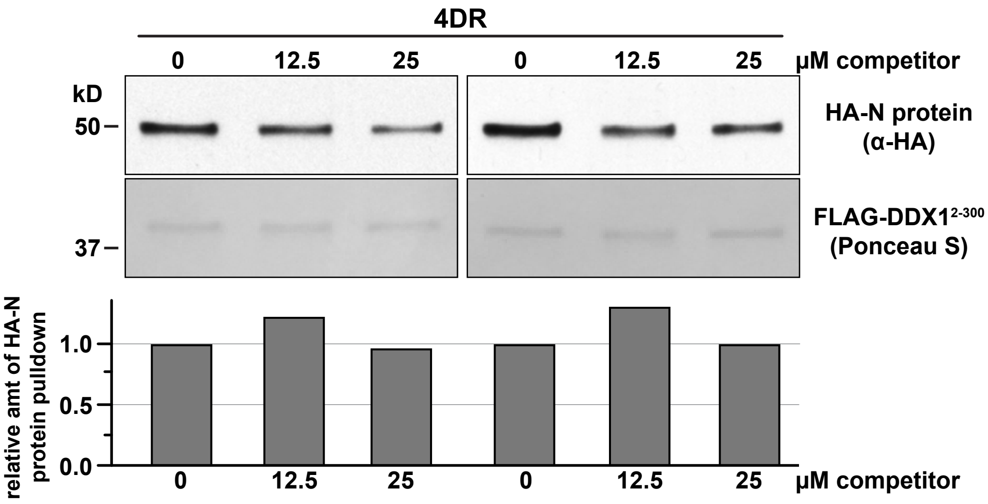

Figure S3. The 4DR peptide competes with full length N-protein for binding to DDX1$^{2\text{-}300}$.
A) Schematic representation of the N protein SR region and the serine (S) to aspartate (D) mutation (4DR) construct tested in the direct competition assay as diagrammed in Figure 5B.
B) Ponceau S and anti-HA tag western blot analyses of the in vitro binding between HA-N protein and FLAG-DDX1$^{2\text{-}300}$ in the presence or absence of the 4DR peptide competitor. The relative amount of HA-N pulldown normalized to the Ponceau S loading control for each condition is graphed with the no peptide controls set to 1.
